# Person-to-Person Transmission of Nipah Virus in a Bangladeshi Community

**DOI:** 10.3201/eid1307.061128

**Published:** 2007-07

**Authors:** Emily S. Gurley, Joel M. Montgomery, M. Jahangir Hossain, Michael Bell, Abul Kalam Azad, Mohammed Rafiqul Islam, Mohammed Abdur Rahim Molla, Darin S. Carroll, Thomas G. Ksiazek, Paul A. Rota, Luis Lowe, James A. Comer, Pierre Rollin, Markus Czub, Allen Grolla, Heinz Feldmann, Stephen P. Luby, Jennifer L. Woodward, Robert F. Breiman

**Affiliations:** * ICDDR,B, Dhaka, Bangladesh; † Centers for Disease Control and Prevention, Atlanta, Georgia, USA; ‡ Ministry of Health and Family Welfare, Dhaka, Bangladesh; § Public Health Agency of Canada, Winnipeg, Manitoba, Canada; ¶ University of Manitoba, Winnipeg, Manitoba, Canada; # University of Texas School of Public Health, Houston, Texas, USA

**Keywords:** Nipah virus, disease outbreak, disease transmission (horizontal), Bangladesh, research

## Abstract

An encephalitis outbreak was investigated in Faridpur District, Bangladesh, in April–May 2004 to determine the cause of the outbreak and risk factors for disease. Biologic specimens were tested for Nipah virus. Surfaces were evaluated for Nipah virus contamination by using reverse transcription–PCR (RT-PCR). Thirty-six cases of Nipah virus illness were identified; 75% of case-patients died. Multiple peaks of illness occurred, and 33 case-patients had close contact with another Nipah virus patient before their illness. Results from a case-control study showed that contact with 1 patient carried the highest risk for infection (odds ratio 6.7, 95% confidence interval 2.9–16.8, p<0.001). RT-PCR testing of environmental samples confirmed Nipah virus contamination of hospital surfaces. This investigation provides evidence for person-to-person transmission of Nipah virus. Capacity for person-to-person transmission increases the potential for wider spread of this highly lethal pathogen and highlights the need for infection control strategies for resource-poor settings.

## Abstract 2

Transmission of this virus highlights the need for infection control strategies for resource-poor settings.

Nipah virus was first identified as the pathogen responsible for outbreaks of encephalitis in Malaysia and Singapore from October 1998 to June 1999 (*1*–*6*). Fever (97%), headache (61%), and reduced consciousness (55%) were the most common symptoms in Malaysia; case-fatality rate was 40% (*7*). Most case-patients lived on pig farms (95% in Malaysia) (*1*) or worked in abattoirs (100% in Singapore) (*4*,*8*). Serologic and reverse transcription–PCR (RT-PCR) testing of blood and urine from pteropid fruit bats in Malaysia and Cambodia showed Nipah virus infection, which suggested that these animals were reservoir hosts (*9*–*11*). During this outbreak, Nipah viruses were also isolated from human respiratory secretions and urine (*2*); however, 2 studies did not find evidence of nosocomial transmission (*12*,*13*).

Subsequent investigations in India and Bangladesh have suggested that Nipah virus may have been transmitted from person to person. During an outbreak in Siliguri, India, in 2001, 45 (75%) of 60 patients, many of them healthcare workers, had a history of hospital exposure to patients infected with Nipah virus (*14*). A case-control study conducted during an outbreak in Meherpur District, Bangladesh, in 2001 showed that persons who lived with or cared for patients during the patient’s illness were more likely to become infected with Nipah virus, and patients were more likely to have reported touching secretions of other patients; however, this finding could not be differentiated from common environmental exposures (*15*). During an outbreak in Rajbari District, Bangladesh, in January 2004, case-patients were more likely than controls to have had contact with another patient with Nipah virus illness (*16*). Pteropid bats were also suspected to be the reservoir for the virus in Bangladesh (*9*–*11*,*15*).

On April 5, 2004, the ICDDR,B and the Institute for Epidemiology and Disease Control Research were alerted to a cluster of 5 persons with fever, headache, confusion, and loss of consciousness in Faridpur District, in western Bangladesh. Nipah virus was the suspected cause of the outbreak and an investigation began on April 6, 2004. Investigation goals were to identify a reservoir host(s), define the magnitude of the epidemic, and determine the principal modes of transmission. This report addresses the last 2 goals.

## Methods

### Finding and Defining Cases

We defined suspected case-patients with Nipah virus illness as persons with fever and altered mental status (serious illness) residing or working in the outbreak area or persons who had fever and cough or headache (mild illness) and who were contacts of patients with Nipah virus infection or resided in the outbreak area. Suspected case-patients were identified by visiting area hospitals, conducting door-to-door visits to all homes in the affected area, and tracing contacts of patients with Nipah virus illness. A history of illness and general information about exposures were obtained for each suspected case-patient. Friends and relatives of deceased case-patients served as proxy informants for interviews, and guardians were included in interviews of children <13 years of age. All those who died in the outbreak areas during this time were considered suspected case-patients.

A probable case-patient was defined as a patient with fever and mental status changes who lived or worked in the same village as a confirmed case-patient and from whom either serum or cerebrospinal fluid (CSF) was not available (i.e., because the patient died before specimen could be collected) or from whom a negative result was obtained from a sample collected <10 days after illness onset but collection of subsequent specimens was impossible (*17*). A laboratory-confirmed case of Nipah virus infection was defined by evidence of acute infection shown by immunoglobulin M (IgM) to Nipah virus in serum or CSF. To evaluate the possibility of asymptomatic infections, we asked persons with a history of close contact with a patient with Nipah virus–like illness to provide a blood specimen for serologic testing (*7*,*13*).

### Specimen Collection and Laboratory Testing

Acute-phase blood specimens, throat swabs, saliva, and urine were collected from persons with suspected cases. When possible, hospitalized patients underwent lumbar puncture and chest radiography. Convalescent-phase blood specimens were collected from all persons with suspected cases >10 days after illness onset. Acute- and convalescent-phase serum and CSF were tested with an IgM capture enzyme immunoassay (EIA) for IgM and an indirect EIA for IgG by using Nipah virus (Malaysian prototype) antigen S (*18*) at the Centers for Disease Control and Prevention in Atlanta, Georgia, USA. Acute-phase serum, CSF, throat swabs, saliva, and urine were also tested by RT-PCR for viral RNA. RNA was extracted from specimens by using the acid guanidinium–phenol method (*19*). RT-PCR was performed by using a primer set to detect the nucleocapsid gene as described (*20*), the Superscript One Step RT-PCR Kit (Invitrogen, Carlsbad, CA, USA), and standard reaction conditions (*21*). Primers used were NVNF-4: 5′-GGA GTT ATC AAT CTA AGT TAG-3′ and NVNBR4: 5′-CAT AGA GAT GAG TGT AAA AGC-3′. PCR products were subjected to electrophoresis on 2% agarose gels and visualized by staining with ethidium bromide. Positive PCR results were confirmed by sequence analysis of PCR products.

### Case–Control Study

We conducted a case–control study to identify risk factors for transmission of Nipah virus infection. Persons meeting either the probable or confirmed case definition were enrolled as case-patients. All healthy persons including household members and neighbors (residing within 150 m of a case household), were eligible for participation as control participants. Controls were randomly selected from a list of names generated by a census of all households in the affected community and matched to a case-patient (6:1) by sex and age (± 2 years; all case-patients and controls were >4 years of age). They were given a 2-part questionnaire in Bengali. The first part focused on environmental exposures and established whether the participant had had contact with a specific probable or confirmed case-patient(s). The second part focused on types of contact with specific case-patients to elucidate possible modes of transmission from person to person. Proxy interviews were conducted with guardians and companions for case-patients who had died or who were unable to respond and for all case-patients and controls <13 years of age.

We calculated odds ratios (ORs) and 95% confidence intervals (CIs) by using conditional univariate logistic regression that accounted for matched enrollment of case-patients and controls (*22*). To evaluate independent risk factors, we tested all variables with a p value <0.1 from univariate analyses in conditional stepwise forward multivariate logistic regression. Associations were considered statistically significant if p value was <0.05. All statistical analyses were performed with SAS version 9.0 (SAS Institute Inc., Cary, NC, USA).

### Environmental Study

In early May 2004, 5 weeks after the outbreak was first recognized, environmental surfaces believed to have a high risk for contamination with Nipah virus were selected for sampling. These included surfaces within hospitals where Nipah virus patients received care, surfaces within homes of confirmed case-patients, trees where bats foraged, date palm sap collection pots, and fruits that may have been in contact with fruit bats. Surfaces were rolled with sterile, cotton-tipped applicators, which were stored in 500 μL Dulbecco modified Eagle medium supplemented with an antibiotic-antimycotic solution. Viral RNA was extracted from 140 μL of the resulting suspension by using a Viral RNA Minikit (QIAGEN, Mississauga, Ontario, Canada) and eluted in a volume of 50 μL. Nipah virus RNA was detected by using the LightCycler RNA Amplification Kit SYBR Green I (Roche, Laval, Quebec, Canada) with primers for the nucleoprotein gene (NPF: 5′-ATCAATCGTGGTTATCTTGAAC-3′ and NPR: 5′CCTCTTCGTCGACATCTTGATC-3′) and with thermocycling and real-time detection performed on a SmartCycler II reaction block (Cepheid, Sunnyvale, CA, USA). Positive results were later confirmed by direct sequencing of amplified products, and sequences were compared with those obtained from patient samples (*23*).

### Participants and Ethical Considerations

Because this was an outbreak investigation, protocols did not undergo formal institutional review. The Bangladesh Ministry of Health and Family Welfare requested this investigation and reviewed and approved all protocols. Participation in these studies was strictly voluntary and informed consent was obtained from all participants; for those <18 years of age, individual and parental consent were obtained.

## Results

### Defining Cases

We identified and collected specimens from 210 suspected case-patients, of whom 32 had fever and altered mental status. Thirty-six case-patients were identified, including 4 who had a febrile illness without altered mental status. Twenty-three (64%) were laboratory confirmed and 13 (36%) were classified as probable; 27 (75%) died. Serum specimens were available from 27 of 36 case-patients, 4 of which had no detectable antibodies to Nipah virus. These specimens were collected <6 days after illness onset. All 4 patients died before a second specimen could be obtained; therefore, they were classified as probable case-patients. Nine patients who had encephalitis-like illnesses and who resided in the outbreak area died before diagnostic specimens could be collected; they were also classified as probable case-patients. No asymptomatic cases of Nipah virus infection were documented in contacts of Nipah virus patients who consented to provide a blood specimen (n = 20). Results of sequencing RT-PCR products from throat swab, saliva, and urine samples of 9 patients were consistent with serologic data, which indicated that Nipah virus was the etiologic agent of this outbreak (*23*).

### Contact Tracing

**Figure 1:**
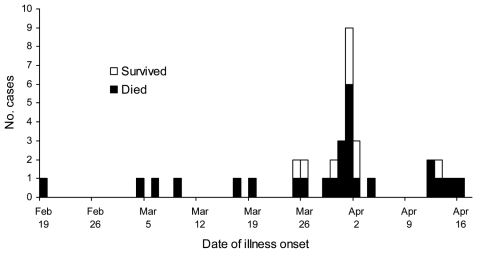
Dates of illness onset during a Nipah virus outbreak in Faridpur District, Bangladesh, 2004.

**Figure 2:**
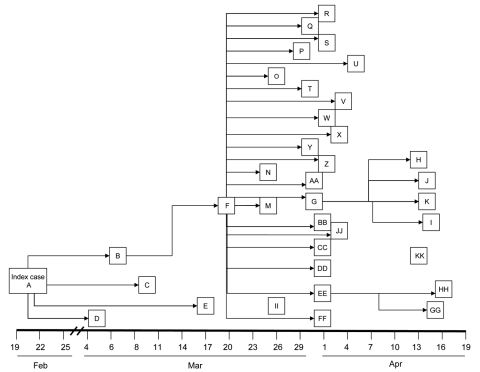
Chain of person-to-person transmission with dates of onset of illness during a Nipah virus outbreak, Faridpur District, Bangladesh, 2004. Letters identify individual patients. Patients KK and II had no known contact with any ill patient before their illness.

Probable and confirmed case-patients were identified in 7 villages in Faridpur District; dates of onset of illness ranged from February 19 to April 17, 2004 (Figure 1). Thirty-three (92%) of the 36 case-patients had had close contact with another ill person before they became ill (Figure 2). Five cases (patients A, B, F, G, and EE) appeared to be associated with secondary and tertiary person-to-person transmission of Nipah virus. We present some examples of close contact.

Four of Patient A’s caregivers who resided in his village became ill after their contact with him; they were his mother (patient D), his son (patient E), his aunt (patient B), and a neighbor (patient C). They became ill 15–27 days after patient A became ill. Patient B received care from her brother (patient F), who lived in a village ≈30 minutes from her village and who became ill 13 days after patient B.

Patient F became ill after he had returned to his village. As shown in Figure 2, 22 (61%) of 36 cases in this outbreak had contact with patient F before they became ill. Patient F was a local religious leader. Many persons in his family and his followers had close contact with him during his illness. Eight (80%) members of his 10-person household became infected with Nipah virus 6–13 days after his illness onset. Two of patient F’s brothers (patients T and U), both of whom lived ≈2 hours away, visited him for 6 hours on the day he died. They developed serious illness 7 (patient T) and 11 days (patient U) days later, and both died. Patients Y (patient F’s daughter) and Z (patient F’s son-in-law), who lived 1 hour away from patient F, became ill ≈1 week after 1 multiple-hour visit with patient F late during his illness; this was their only reported contact with a Nipah virus patient. In 11 other close contacts of patient F, including family and religious followers, Nipah virus infection developed 6–14 days after his illness onset.

Patient G, a follower of patient F, moved to his family’s house in an adjacent village to receive care after becoming ill. Patient H became ill ≈9 days after physically supporting patient G while walking to patient G’s family’s house. A rickshaw driver, patient I, who helped carry and transport patient G to the hospital as his condition deteriorated, became sick 10 days after this exposure and later died. Patient G’s father (patient J) and sister-in law (patient K) cared for him during his illness; both became severely ill 2 weeks after patient G’s illness onset; only patient K survived.

Patient GG visited his wife and daughter, both hospitalized as suspected Nipah virus patients, at the local healthcare facility. Patient GG spent the night in the hospital and shared a bed with patient EE (a common practice in Bangladesh), who was a male friend, and a suspected Nipah virus case-patient. Although tests on patient GG’s wife and daughter did not detect Nipah virus infection, patient EE’s infection was laboratory confirmed. Patient GG became ill 10 days after this contact and died.

Patients II and KK had no known contact with any ill patient before their illness and were distinct from other case-patients in that they were not friends, relatives, or followers of patient F and lived outside the affected villages. No cases of Nipah virus illness among healthcare workers were reported to authorities during this outbreak.

### Case–Control Study

Thirty-four of the 36 case-patients were enrolled in the study and matched to 6 controls each (n = 204) by age and sex (Table). Two case-patients (patients JJ and KK; Figure 2) were not included in the study because of logistic constraints. Patient JJ was away from her home when the questionnaire was administered. Patient KK was a policeman who had 2 residences and traveled frequently while on duty; we were therefore unable to identify appropriate proxies for his interview.

**Table:** Characteristics of 34 case-patients infected with Nipah virus in case-control study, Bangladesh, April–May 2004

Characteristic	No. (%) case-patients
Sex	
Male	20 (58.8)
Female	14 (41.2)
Age group, y	
1–15	4 (11.8)
16–24	1 (2.9)
25–40	19 (55.9)
41–60	9 (26.5)
>60	1 (2.9)
Adults vs. children	
<15 y of age (children)	4 (11.8)
>15 y of age (adults)	30 (88.2)

Ten variables were significantly associated with Nipah virus infection in univariate analysis (Appendix Table 1). Having had close contact (touching or receiving a cough or sneeze in the face) with patient F placed a person at greatest risk of acquiring Nipah virus infection (OR 6.7, 95% CI 2.9–16.8, p<0.001). Having had any contact with someone who later died, had a fever, was unconscious, or had respiratory difficulties was also associated with illness. Having avoided any contact with someone who later died was negatively associated with illness. Having had a household member harvest date palm sap was the only environmental exposure associated with an increased risk for infection. Having visited the home village of the index case-patient was also associated with illness. However, the only exposure variable that remained significant in multivariate analysis was having had contact with patient F (OR 5.6, 95% CI 1.79–17.24, p = 0.003).

Univariate analysis of risk factors specifically associated with types of contact with patient F was conducted. Fourteen of 42 exposure variables were associated with illness ( Appendix Table 2). Having had close body contact and having spent longer periods of time with patient F were associated with illness. Having kept a certain distance from patient F and having washed hands after contact with him were negatively associated with illness ( Appendix Table 2). Despite multivariate analysis of risk factors associated with type of contact with patient F, insufficient sample size (n = 50) resulted in overfitting of the model and spurious results.

### Environmental Study

A total of 468 environmental specimens were collected by swabbing; 137 from walls, bed frames, mattresses, and floors of 2 Faridpur hospitals; 57 from surfaces and utensils of case-patient residences; 150 from trees where bats forage and from fruits; 98 from bat excreta; and 26 from other sites. Eleven positive specimens were collected from the surrounding wall and bed frame where a confirmed case-patient (patient Z) had been hospitalized on April 6, ≈5 weeks before environmental samples were collected. No other patients with encephalitis were known to have used that bed after patient Z’s hospitalization. The wall and bed frame were visibly soiled, and hospital staff reported that they had not been cleaned since the outbreak. Samples from these areas showed evidence of Nipah virus RNA. Sequences of PCR products were identical to sequences of Nipah viruses isolated from patient HH during the outbreak (*18*). No samples from case-patient residences, bat-feeding sites, or fruits were positive.

## Discussion

This investigation provides compelling evidence for person-to-person transmission of Nipah virus. Exposure histories of infected patients and the epidemiologic curve, which demonstrates multiple peaks of illness onset during this outbreak, suggest that Nipah virus was transmitted by person-to-person contact. Contact tracing documented Nipah virus illness after brief, yet close contact, with other persons infected with Nipah virus. Findings from the case-control study, which showed a 6-fold increased risk for infection for those who reported contact with patient F, a negative association with illness after handwashing, and specific exposures to ill persons linked to transmission, confirm that exposure to ill persons spread the outbreak.

Person-to-person transmission of Nipah virus is biologically plausible. Other paramyxoviruses that infect humans, including human parainfluenza viruses 1–4, measles virus, and mumps virus, are also transmitted from person to person. Nipah virus has been isolated from human respiratory secretions, including those of cases from this outbreak (*2*,*23*). Furthermore, we identified that direct exposure to respiratory secretions of patients with Nipah virus illness was associated with infection during this outbreak.

The number of villages affected by the outbreak increased as persons traveled in and out of the affected areas to visit family members. This movement led to new infections in caregivers from other villages and increased the number of villages affected. Similar to transmission of severe acute respiratory syndrome (*24*), transmission of Nipah virus infection was not associated with all case-patients; however, 1 case-patient, patient F, was associated with 22 subsequent Nipah virus infections. Although host biologic factors may have resulted in increased viral shedding, leading to higher attack rates, the social status of patient F in the community enabled closer contact with more persons during his illness and more opportunity to transmit infection.

During the outbreak in Siliguri, India, 33 healthcare workers and hospital visitors became ill after exposure to hospitalized patients with Nipah virus illness, suggesting nosocomial infection (*14*). In Malaysia and Singapore, contact with pigs was associated with infection; healthcare worker studies showed that the risk for nosocomial transmission was low (*6*,*8*,*12*,*13*,*25*). Absence of person-to-person transmission in Malaysia and Singapore could be due to differences in patient care practices, host susceptibility factors, or strain variation (*23*).

Detection of Nipah virus RNA on hospital surfaces demonstrates that infected patients shed virus into the environment, which could provide an opportunity to transmit Nipah virus to others. However, how long the virus will remain infectious in the environment is not known, and no evidence from this investigation indicates that type of transmission occurred. Despite the absence of healthcare worker infection during this outbreak, enhanced infection control practices, such as patient isolation and use of gloves and masks, likely had little protective effect because they were not implemented until late in the outbreak. This outbreak provides evidence that 1 person (patient GG) was infected during a hospital visit while sharing a bed with a confirmed case-patient. Nosocomial transmission of Nipah virus was reported during the outbreak in Siliguri, India (*14*). Efforts are needed to develop and disseminate reasonable guidelines for infection control and prevention for healthcare facilities and communities in resource-poor settings, especially when one considers our finding that handwashing prevents disease transmission.

Transmission of Nipah viruses to humans during this outbreak appears to have been bimodal. Fruit bats continue to be the only identified primary reservoir for the virus (*9*–*11*,*15*). In contrast with the Malaysia and Singapore experience, no intermediate hosts have been identified in Bangladesh (*15*). During this outbreak, the introduction of virus into human(s) from an unknown initial source appears to have been followed by person-to-person transmission. Three case-patients had no known contact with a sick patient before onset of illness; these case-patients may have been infected through exposure to virus-contaminated bat saliva, urine, or feces or through contact with some unknown intermediate host. These cases provide further evidence that sporadic cases of Nipah virus infection continue to occur in Bangladesh (*26*).

Selection of probable case-patients in this study could have been biased toward finding person-to-person transmission because probable case-patients by definition lived in the same area as confirmed case-patients, which increased the likelihood that they had had contact with one another. To rule out the possibility that this had an effect on our case-control study findings, we analyzed our data by using only confirmed cases; there were no major differences in findings compared with analysis that used confirmed and probable cases. Our findings are also limited by recall bias. Family and friends were often asked to provide information about a deceased patient weeks after their illness (data for 5 patients were collected >1 month after illness onset). When possible, medical records were used to supplement patient reports of illness history, but often these records were incomplete or nonexistent. However, the investigation began just days after onset of illness in most patients, which provided for optimal recall of events. In addition, case-patients or their proxies might have more carefully considered their exposures than controls. However, all community members were aware of, and concerned by, the outbreak, and we believe it is unlikely that any control did not remember their exposure to case-patients in their community.

Capacity for person-to-person transmission increases the risk for wider spread of this highly lethal pathogen. In an impoverished, densely populated country such as Bangladesh, a lethal virus could rapidly spread before effective interventions are implemented. This spread would provide the seed for a substantial regional or global public health problem and highlights the need for local surveillance, outbreak detection and response, and rapid laboratory diagnostics. Sustained, long-term research is needed to characterize the reservoir of the virus and mechanisms for animal-to-animal, animal-to-human, and human-to-human transmission; clarify climatologic and other environmental factors linked to transmission; and define viral epitopes potentially linked to virulence and transmission. Effective infection control practices appropriate for resource-constrained healthcare systems and communities are urgently needed.

## Supplementary Material

Appendix Table 1 Exposures and activities associated with Nipah virus infection, Bangladesh, April-May 2004*

Appendix Table 2 Exposures to patient F and association with Nipah virus infection, Bangladesh, April-May 2004*
