# Supplementary material for: Person-to-Person Transmission of Nipah Virus in a Bangladeshi Community
Source: Emerg Infect Dis. 2007 Jul;13(7):1031–7. doi: 10.3201/eid1307.061128 (PMC2878219; doi:10.3201/eid1307.061128)
Supplement: Appendix Table 1 — Exposures and activities associated with Nipah virus infection, Bangladesh, April-May 2004* [file 06-1128_appT1-s1.pdf]

Appendix Table 1. Exposures and activities associated with Nipah virus infection, Bangladesh, April–May 2004*

Exposure or activity	No. (%) with reported exposure or activity: Case-patients (n = 34)	Controls (n = 204)	OR (95% CI)†	p value
Bat exposures				
Touched	0	4 (2)	1.1 (0.0–10.6)	1.00
Ate	0	1 (0.5)	6.0 (0.0–234.0)	1.00
Observed an increase in no. of fruit bats in or around house	0	4/202 (2)	1.14 (0.0–9.089)	1.00
Environmental exposures				
Ate date palm sap	11/31 (35)	102 (50)	0.51 (0.198–1.26)	0.163
Harvested date palm sap	2/33 (6)	3 (2)	4.0 (0.33–34.9)	0.30
Anyone in household harvested date palm sap	5 (15)	8/203 (4)	4.3 (1.0–17.1)	0.049‡
Touched or drank directly from a date palm sap collection bowl	6/33 (18)	16 (8)	2.5 (0.74–7.65)	0.15
Ate partially eaten fruit from ground	6 (18)	32 (16)	1.2 (0.35–3.28)	0.94
Travel history				
Visited village of patient F	26/32 (81)	156 (76)	1.3 (0.49–4.07)	0.747
Visited village of index case	15/33 (45)	34 (17)	4.04 (1.76–9.31)	0.0008‡
Exposure to human illness				
Touched person who was unconscious	19/32 (59)	74/200 (37)	2.6 (1.15–6.16)	0.019‡
Touched person who later died	23/31 (74)	73/197 (37)	5.5 (2.14–16.3)	0.0001‡
Touched person who had seizures	4/29 (14)	39/199 (20)	0.69 (0.16–2.29)	0.75
Touched person who had a fever	27/33 (82)	108/202 (53)	3.9 (1.51–12.01)	0.003‡
Touched person who had respiratory difficulties	19/32 (59)	65/197 (33)	3.3 (1.4–8.27)	0.0051‡
In the same room (not touching) with person who				
Was unconscious	24/33 (72)	120/200 (60)	1.9 (0.79–4.84)	0.183
Later died	25/32 (78)	112/200 (56)	3.1 (1.2–9.13)	0.0159‡
Had seizures	6/29 (21)	60/198 (30)	0.62 (0.19–1.66)	0.438
Had a fever	29 (85)	148/203 (73)	2.15 (0.77–7.43)	0.174
Had respiratory difficulties	20/33 (61)	111/200 (56)	1.19 (0.54–2.74)	0.77
No contact with person who				
Was unconscious	9/33 (27)	71/200 (36)	0.65 (0.25–1.55)	0.404
Later died	7/32 (22)	82/201 (41)	0.37 (0.13–0.96)	0.039‡
Had seizures	24/30 (80)	130/199 (65)	2.1 (0.76–6.52)	0.187
Had a fever	5 (15)	54/203 (27)	0.48 (0.14–1.33)	0.19
Had respiratory difficulties	13/33 (39)	88/201 (44)	0.87 (0.38–1.95)	0.87
Contact with a specific case(s)§¶				
Patient A	4 (12)	17 (8)	1.5 (0.34–4.8)	0.70
Patient B	8 (24)	8 (4)	6.5 (2.0–21.2)	0.001‡
Patient F	18 (53)	32 (16)	6.7 (2.9–16.8)	<0.0001‡#
Patient G	3 (9)	7 (3)	2.9 (0.43–15.1)	0.31
Patient EE	2 (6)	15 (7)	0.79 (0.09–3.5)	1.0
Patient II	0	5 (2)	0.89 (0.0–6.5)	0.93
Smoking history				
Currently smoke	13 (38)	61 (30)	1.9 (0.61–6.4)	0.32
Share cigarettes, biri, hukka, or pipe	10/33 (30)	45 (22)	1.9 (0.61–5.9)	0.31

*Values are no. subjects responding affirmatively/total no. responding (%) unless otherwise indicated. OR, odds ratio; CI, confidence interval.
†Exact method using univariate conditional logistic regression.
‡Included in multivariate conditional logistic regression analysis.
§Contact was defined as seeing, talking, eating or drinking, touching, and providing care.
¶Contact with ≥1 of the 34 probable or confirmed case-patients (selected few shown in table).
#Remained in final multivariate model.
