# Supplementary material for: Person-to-Person Transmission of Nipah Virus in a Bangladeshi Community
Source: Emerg Infect Dis. 2007 Jul;13(7):1031–7. doi: 10.3201/eid1307.061128 (PMC2878219; doi:10.3201/eid1307.061128)
Supplement: Appendix Table 2 — Exposures to patient F and association with Nipah virus infection, Bangladesh, April-May 2004* [file 06-1128_appT2-s2.pdf]

Appendix Table 2. Exposures to patient F and association with Nipah virus infection, Bangladesh, April–May 2004*

Exposure	No. (%) with reported exposure or activity		OR (95% CI)†	p value
	Case-patients (n = 18)	Controls (n = 32)		
Contact with patient F				
During illness	16 (89)	31 (97)	0.27 (0.004–5.46)	0.583
Day of death	13 (72)	15 (47)	2.9 (0.74–12.90)	0.149
After death	17 (94)	22 (69)	7.5 (0.903–354.68)	0.069
Patient F activity				
Coughed while nearby	15/17 (88)	17/25 (68)	3.4 (0.559–38.15)	0.252
Unconscious while nearby	15 (83)	30/31 (97)	0.17 (0.003–2.365)	0.268
Vomited while nearby	8 (44)	9/30 (30)	1.8 (0.465–7.39)	0.481
Proximity to patient F				
Always >6 m	0 (0)	2 (6)	0.72 (0.0–9.518)	0.809
Always >1.5 m but <6 m	2 (11)	20 (63)	0.08 (0.008–0.427)	0.008
Always >0.5 m but <1.5 m	4 (22)	3 (9)	2.7 (0.398–21.04)	0.4015
Sometimes <30 cm (1 foot)	1 (6)	5 (16)	0.324 (0.006–3.267)	0.570
Sometimes <15 cm (0.5 feet)	7 (39)	3 (9)	5.9 (1.106–47.79)	0.035
Sometimes touching	6 (33)	1 (3)	14.6 (1.53–735.12)	0.013
Length of time spent with patient F				
<10 min	2 (11)	10 (31)	0.28 (0.026–1.59)	0.205
10–15 min	2 (11)	9 (28)	0.33 (0.03–1.89)	0.298
30 min	0	9 (28)	0.11 (0.0–0.766)	0.022
1–1.5 h	0	7 (22)	0.154 (0.0–1.122)	0.067
3–4 h	2 (11)	1 (3)	3.76 (0.183–235.8)	0.583
>4 h	16 (89)	6 (19)	31.2 (5.35–351.68)	<0.0001
Specific interactions with patient F				
Received sneeze/cough in face from patient	4/13 (31)	2 (6)	6.3 (0.766–80.81)	0.097
Ate with patient	4 (22)	2 (6)	4.2 (0.524–51.05)	0.228
Shared same plate/bowl/cup/glass	6 (33)	1 (3)	8.5 (0.749–449.8)	0.101
Fed patient with a spoon or cup	10 (56)	1 (3)	35.3 (4.064–999.9)	<0.0001
Fed patient with hands	7 (39)	1 (3)	18.5 (2.02–916.63)	0.004
Talked with patient	11 (61)	4 (13)	10.3 (2.25–59.31)	0.0011
Held patient's hands	13 (72)	6 (19)	10.6 (2.443–55.26)	<0.0006
Touched patient's face	12/17 (71)	4 (13)	15.5 (3.18–96.86)	0.0001
Shared a bed with patient	2 (11)	0	4.5 (0.341+ Inf)	0.25
Helped patient walk, sit, or stand	8 (44)	4 (13)	5.4 (1.15–30.18)	0.03
Lifted or carried patient	2 (11)	2 (6)	1.9 (0.12–27.8)	0.91
Cleaned patient's hands with a cloth or clothing	5 (28)	0	14.9 (1.89 + Inf)	0.0081
Cleaned patient’s face with a cloth or clothing	4 (22)	0	11.0 (1.29 + Inf)	0.027
Wiped patient's face with hands	3 (17)	0	7.6 (0.775 + Inf)	0.083
Wiped patient's nose/mouth with hands	3 (17)	0	7.6 (0.775 + Inf)	0.083
Helped patient change clothes	1 (6)	0	1.78 (0.046 + Inf)	0.72
Helped patient use the toilet	2 (11)	0	4.5 (0.41+ Inf)	0.249
Cleaned feces from patient's body	0	0	Undefined	NA
Changed patient's bed linens	0	0	Undefined	NA
Washed patient's clothes	1 (6)	0	1.78 (0.046 + Inf)	0.72
Washed patient's bed linens	0	1 (3)	1.78 (0.0–69.3)	1.00
Dried out patient's mouth after death	0	1 (3)	2.3 (0.0–89.14)	1.00
Always/sometimes kept face covered while near patient	0	0	Undefined	NA
Activity after contact with patient F				
Washed hands	2/17 (12)	15/32 (47)	0.196 (0.03–0.895)	0.032

*Values are no. of subjects responding affirmatively/total no. responding (%) unless otherwise indicated. OR, odds ratio; CI, confidence interval; NA, not available.

†Exact method using univariate conditional logistic regression. + Inf, positive infinity, an unknown upper limit for the CI.
